# Perceived Masculinity Predicts U.S. Supreme Court Outcomes

**DOI:** 10.1371/journal.pone.0164324

**Published:** 2016-10-13

**Authors:** Daniel Chen, Yosh Halberstam, Alan C. L. Yu

**Affiliations:** 1 Institute for Advanced Study, Toulouse School of Economics, Toulouse, France; 2 Department of Economics, University of Toronto, Toronto, Ontario, Canada; 3 Phonology Laboratory, Department of Linguistics, University of Chicago, Chicago, Illinois, United States of America; Macquarie University, AUSTRALIA

## Abstract

Previous studies suggest a significant role of language in the court room, yet none has identified a definitive correlation between vocal characteristics and court outcomes. This paper demonstrates that voice-based snap judgments based solely on the introductory sentence of lawyers arguing in front of the Supreme Court of the United States predict outcomes in the Court. In this study, participants rated the opening statement of male advocates arguing before the Supreme Court between 1998 and 2012 in terms of masculinity, attractiveness, confidence, intelligence, trustworthiness, and aggressiveness. We found significant correlation between vocal characteristics and court outcomes and the correlation is specific to perceived masculinity even when judgment of masculinity is based only on less than three seconds of exposure to a lawyer’s speech sample. Specifically, male advocates are more likely to win when they are perceived as less masculine. No other personality dimension predicts court outcomes. While this study does not aim to establish any causal connections, our findings suggest that vocal characteristics may be relevant in even as solemn a setting as the Supreme Court of the United States.

## Introduction

Voice-based first impressions can be formed rapidly with very brief exposure (less than half a second of speech [1–4]) and such impressions often are associated with subsequent behavior of the perceiver [5–7]. For example, voice-based personality judgments are associated with mate selection [8], leader election [9, 10], housing options [11], consumer choices, and jury decision [12]. Although researchers have demonstrated how vocal perception influences the communication process [13], it remains unclear whether such influences find resonances in a communicative setting like oral arguments at the Supreme Court of the United States (SCOTUS), where subtle biases have consequences for major policy outcomes. To be sure, previous studies suggest a significant role for linguistic cues in the court room [12, 14, 15], yet none has identified a definitive connection between voice perceptions and actual court outcomes.

*A priori*, there are many reasons why inferences from voice should not play an important role in Supreme Court decisions. From a rational perspective, information about the advocate should override any first impression. From an ideological perspective, court outcomes are largely predetermined. From a judge’s legal perspective, decisions are justified not in terms of the advocate’s voice but in terms of the legal content of the argument. And from an economic perspective, correlations between malleable advocate characteristics and high-stakes outcomes in the United States Supreme Court should not persist as law firms and advocates are likely to adjust their behavior to eliminate such correlations.

At the same time, from a behavioral perspective, however, it has been repeatedly shown that the way one speaks reveals a lot about one’s personality, level of confidence, as well as ethnicity, socio-economic circumstances, geographic background, sexuality, and ideological stance [8, 16–18]. The identification of African American speakers can be made even on the basis of the single word “hello” [11]. The percept of gay male speech and/or feminine male speech is linked to vowel formant structure [19], pitch [20] and the length and quality of /s/ [21, 22]. The released variant of word-final /t/ may be used as a resource of constructing nerd identity among female nerds [23], learnedness among Orthodox Jewish men [24], gayness [25] and articulateness among US politicians [26]. To be sure, listeners’ interpretations of the meanings behind these linguistic cues might vary according to the listener’s level of experience with different speech varieties [27] and the identity of the speaker [26]. Nonetheless, even when visual cues are present, potential employers rely more on voice-based impressions of a job applicant’s competence and intellect in making hiring decisions [28].

In this study, we examine the relationship between how people perceive the voice personalities/attributes of advocates arguing before the court and whether these perceptions can predict real outcomes. To this end, we utilize recordings of oral arguments of the Supreme Court of the United States, which offer a wealth of court decisions that have real world impact. Specifically, we focus on the introductory statement of an oral argument. During an oral argument, counsels representing the competing parties of a case (i.e., the advocates for the petitioner and the respondent) each present their sides to the Justices. As the introductory statement of an advocate’s argument before the court is customarily “Mister Chief Justice, (and) may it please the court”, the corpus of introductory statements we have amassed provides a unique opportunity for examining the effect of speech and language on real world outcomes since the lexical content (the words) being evaluated is identical across speakers. The listeners can therefore focus their judgments on how the words are pronounced, rather than on the word choice of the advocates.

Our empirical strategy is focused on testing models of cognitive bias. To infer the bias, we need to measure perceptions, which are typically unobserved, and how they relate to outcomes. Here, we focused on six dimensions, selected based on previous research on listener’s perceptual evaluations of linguistic variables [18, 29, 30]. These include masculinity, attractiveness, confidence, intelligence, trustworthiness, and aggressiveness. Masculine voices increase perception of dominance and fighting ability among men [31] and they increase attractiveness to women. Vote choices have also been shown to be influenced by perceptions of masculinity and femininity in male faces [32] and judgments about faces are shown to predict the outcomes of actual elections [32, 33]. Vocal attractiveness is often found to be linked to facial attractiveness [34–36]. Judgments of attractiveness are important in everyday interaction as physically attractive people are found to be more persuasive [37] and judged to be more socially desirable and to get better jobs [38]. Confidence, trustworthiness, and aggressiveness are all important aspects of human communication, which can be processed upon one’s very first encounter with an individual [39, 40]. Trustworthiness may, at least partly, influence attribution of competence and might affect voting behavior [33]. It is also an important precursor in the development of cooperation [41] and a fundamental aspect of the legal system [42]. Expressions of confidence have been shown to affect persuasion [43]. Aggressiveness, which indexes a person’s assertiveness, also provides a means to counter the positive orientation of the dimensions considered. A person’s intelligence cannot be observed directly and must be inferred from indirect cues such as voice. Perceived intelligence has been found to affect an individual’s employability [28]. Listeners’ judgments along these dimensions are used as predictors of court outcomes. Given the exploratory nature of this study, it is worth emphasizing at the outset that it is not the goal of this study to advance any claims for any specific causal influence of voice on the SCOTUS outcomes. Rather, we aim to test whether people’s subjective voice-based trait judgments are predictive of the SCOTUS outcomes at all. To the extent that such correlations can be established, future studies will be needed to determine the causal mechanisms behind such relationships.

This article begins with detailing the materials and methodologies used in this study in Section 2. The results are reviewed in Section 3, followed by robustness checks and extensions in Section 4. A discussion of the general findings is given in Section 5.

## Materials and Methods

### Ethics Statement

The study was approved by the Social and Behavioral Sciences Institutional Review Board at the University of Chicago, including a wavier of informed consent as it was determined that the research presents no more than minimal risk to subjects and a waiver of informed consent would not adversely affect the rights and welfare of subjects.

### Stimuli

The stimuli for this study were drawn from oral arguments made in the Supreme Court of the United States between 1998 and 2012. A novel feature of our data is the use of identical 2 to 3 seconds of content delivered at the outset of each argument: “Mr. Chief Justice, (and) may it please the Court”. Our data consist of 1634 oral arguments made by 916 distinct male advocates, where about 80 percent of these advocates argued only once in the Supreme Court.

Oral arguments at the Supreme Court have been recorded since the installation of a recording system in October 1955. The recordings and the associated transcripts were made available to the public in electronically downloadable format by the Oyez Project (http://www.oyez.org/), which is a multimedia archive at the Chicago-Kent College of Law devoted to the Supreme Court of the United States and its work. The audio archive contains more than 110 million words in more than 9000 hours of audio synchronized, based on the court transcripts, to the sentence level.

Oral arguments are, with rare exceptions, the first occasion in the processing of a case in which the Court meets face-to-face in consideration of the issues. Usually, counsels representing the competing parties of a case each has thirty minutes in which to present their side to the Justices. The Justices may interrupt these presentations with comments and questions, leading to interactions between the Justices, the lawyers and, in some cases, the amici curiae, who are not a party to a case but nonetheless offer information that bears on the case not solicited by any of the parties to assist the Court. While oral arguments have been recorded since 1955, with the exception of those between 1998 to 2012, the bulk of the transcripts available on the OYEZ archive at the time this experiment was set up did not identify the speaking turns of individual Justices, referring to them all as “The Court”. The archive has since diarized all recordings.

### Participants

Participants from Amazon MechanicalTurk (AMT) rated the voice clips of the Supreme Court advocates. About half (321) of the 634 distinct participants who completed our survey were female. Two thirds of the participants aged between 20 and 35 years old and one third were older than 35. Likewise, one third indicated they had some college education, whereas one third claimed to have a bachelor’s degree. The median income of those who completed the survey was about 40,000 US dollars. The racial and geographical distribution of the participants broadly reflect that of the US population. The correlation between the share of participants from a given state and the state share of US population is 0.9588. Further descriptive statistics of the AMT participants who participated in this research are presented in Table 1.

**Table 1:** Descriptive Statistics of Survey Participants (N = 634). This table presents descriptive statistics of survey participants who rated audio clips of Supreme Court oral arguments made by male advocates. The data are self-reported by participants before beginning the audio survey.

Participant Characteristic	Frequency	Percent
*Gender*		
Female	321	50.63
Male	313	49.37
*Race*		
African American	58	9.15
American Indian or Native American	4	0.63
Asian	49	7.73
Hispanic or Latino/Latina	39	6.15
Native Hawaiian or Pacific Islander	3	0.47
White	481	75.87
*Age*		
18 to 21	34	5.47
22 to 26	143	22.99
27 to 31	146	23.47
32 to 40	157	25.24
41 to 50	78	12.54
51 or older	64	10.29
*Education*		
Associate’s degree	73	11.51
Bachelor’s degree	216	34.07
Doctoral degree	3	0.47
Graduated high school	61	9.62
Master’s degree	43	6.78
No high school-level education	2	0.32
Professional degree	9	1.42
Some college	218	34.38
Some high school	9	1.42
*Income*		
Between $20,001 to $40,000	196	30.91
Between $40,001 to $60,000	135	21.29
Between $60,001 to $80,000	80	12.62
Less than $20,000	126	19.87
More than $80,000	97	15.30
*Region*		
Midwest	114	17.98
Northeast	133	20.98
South	236	37.22
West	151	23.82

### Procedure

Participants were asked to rate the voice clips of Supreme Court advocates on a scale of 1 to 7 in terms of aggressiveness, attractiveness, confidence, intelligence, masculinity, and trustworthiness. As noted in the Introduction, these six dimensions were selected based on previous research on listener’s perceptual evaluations of linguistic variables [18, 29, 30]. Each voice clip was played aloud once automatically, but participants were allowed to replay the clip as many times as they chose; in another survey variant, each clip was played only once and participants were unable to replay the clip. We discuss this and other survey designs below. The order and polarity of the attributes were randomized across survey participants. For example, masculine would vary vertically along the 6 attributes, and *very masculine* and *not at all masculine* would vary from left to right as bounds on a 7-point scale. The order and the polarity of attribute scales were held fixed for any particular participant to minimize cognitive fatigue. Participants were also asked to predict whether the lawyer would win the case and to rate the quality of the audio recordings.

**Fig 1:**
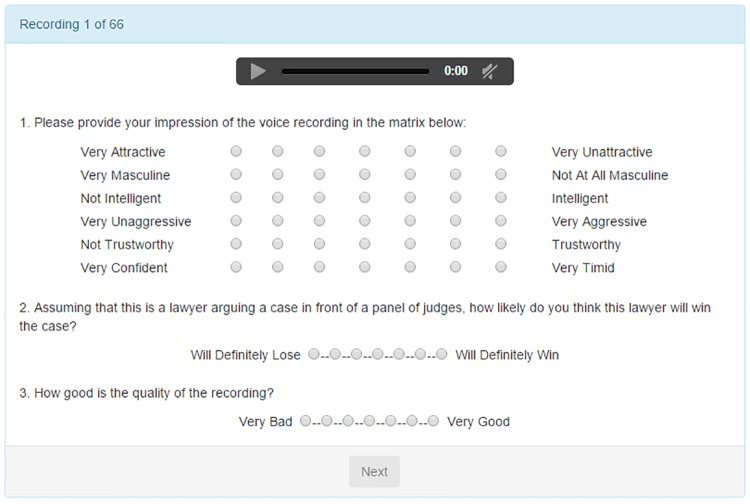
Survey filled by AMT participants. This figure is a screenshot of the survey matrix used by AMT participants to record their impressions of the audio recordings of advocates. The order and polarity of attributes were randomized across participants. Participants were not able to proceed to the next recording without completing the survey matrix and questions.

Each participant rated 66 voice recordings. Of these, 60 were randomly drawn from the audio clip sample pool, and 6 of these were repeated as recordings 61 to 66 to measure the consistency of participant ratings. The participants were asked to use headphones to listen to the recordings. Amici curiae were also rated among the advocates, but are excluded from this study. No information regarding the identity of the speaker or the nature of the case were given to the participants. In Fig 1, we present a screenshot of the survey ratings page. (See S2, S3, and S4 Figs for screenshots of other sections of the task.)

### Analysis

This section lays out the general analytic framework we employed in this study. To operationalize our empirical analysis we begin by constructing a measure of voice-based trait judgments. Let *attribute*_*itw*_ be participant *w*’s perception of a given attribute of advocate *i* in case *t*, where *attribute* refers to any one of the six traits. These untransformed scores (range = 1–7) give more weight to participants who provide more signal amid greater variance in their ratings. Thus, to be conservative, our preferred measure adjusts for cross-participant variability in the cardinality of ratings as well as spread. Formally, for each participant and voice attribute, the normalized rating is given by

$$\begin{array}{c} \hat{attribute}{}_{itw}=\frac{attribute_{itw}-\bar{attribute}{}_{w}}{\sigma {\left(attribute\right)}_{w}}, \end{array}$$ (1)

where $\bar{attribute}{}_{w}$ is the average perception of a given attribute across participant *w*’s advocate ratings and *σ*(*attribute*)_*w*_ is the standard deviation of these ratings. As a result, for each participant *w*, $\hat{attribute}{}_{itw}$ is a continuous measure with mean equals to zero and standard deviation equals to one.

Using these measures, we estimate regression of the following form:

$$\begin{array}{c} case\;outcome_{it}=\alpha +\hat{\text{attribute}}{}_{itw}^{\prime }\beta +{\text{x}}_{itw}^{\prime }\gamma +\varepsilon_{itw}, \end{array}$$ (2)

where the dependent variable is an indicator for whether advocate *i* actually won (= 1) or lost (= 0) case *t*, and the key independent variables denoted by the vector ${\text{attribute}}_{itw}$ are continuous measures of the set of six attributes of the advocate in case *t* as perceived by participant *w*, as well the (normalized) perceived likelihood of winning. Given the regression equation, *β* represents the bias in actual wins associated with advocate traits. The vector ${\text{x}}_{itw}$ is a set of advocate and participant covariates (described in Table 1) that we use to explore the influence of heterogenous perceptions of survey participants on our findings. These covariates include their age, gender, race, income, education and state of residence. To address the correlation in ratings among survey participants, we adjust the standard errors of the regression estimates for clustering at the oral argument level.

For comparison purposes and for robustness, we also show baseline results using the untransformed scores as well as a collapsed version of the data, whereby we match only one voice measure to each oral argument by taking the average rating across participants for a given oral argument. In these regressions we lose variation in perceptions across participants. Broadly, these aggregated regressions mitigate the influence of classical measurement error that typically biases coefficient estimates toward zero. Additionally, using the collapsed data addresses any concern for mechanically increasing power by duplicating the number of oral arguments by the number of ratings per recording (even though we cluster at the recording-level in all regressions). On the other hand, aggregated regressions can lose precision because we also can no longer control for rater-specific correlations across perceptual ratings and participant characteristics. For these reasons, the aggregated regression is generally viewed as too conservative in terms of statistical precision [44]. For sake of completeness, we provide baseline results using the collapsed data as well.

We use the linear probability model (OLS) as our primary estimation method, and show that our results are robust to the use of probit and logistic models. There are two main reasons for this choice. The first is that our objective is to estimate the correlation coefficients between perceived attributes of advocates and case outcomes rather than to develop a forecasting model of case outcomes, and OLS is superior for estimation purposes. And second, probit and logit are not well-suited to the use of regressions with controls for fixed effects (e.g., dummies for lawyer, participant, year of case argued, etc.) because of the incidental parameters problem [45], and our analysis includes many regressions with controls for fixed effects.

## Results

Our procedure produced 33,666 ratings, with approximately 20 ratings for each of the 1634 oral arguments made by male advocates. The total number of observations generated by AMT was 41,844 = 66 ratings x 634 participants. However, ratings of amici curiae as well as ratings by 31 participants that did not vary across recordings were excluded from analysis. The final dataset we use in this paper can be downloaded at: https://figshare.com/s/eede53edfedf12a75c01. Table 2 provides summary statistics of the normalized voice ratings. As expected, the mean normalized rating across participants is approximately zero with a standard deviation of one.

**Table 2:** Summary Statistics of Case Outcome and Trait Judgements of Male Lawyers (N = 33,666). This table presents summary statistics of participant normalized ratings of our sample of 1634 oral arguments. Each observation is an argument by participant rating. *Case Outcome* is an indicator for whether the advocate won the case (= 1) in court or lost (= 0).

Variable	Mean	SD	Min	Max
Case Outcome	0.518	0.500	0.000	1.000
Aggressive	0.002	0.994	−7.261	8.001
Attractive	−0.005	0.992	−8.001	5.701
Confident	0.002	0.993	−4.641	4.172
Intelligent	−0.006	0.999	−8.001	8.001
Masculine	0.014	0.989	−6.308	4.031
Trustworthy	−0.007	0.996	−8.001	8.001
Win	0.000	0.995	−5.787	8.001

Throughout this paper, we refer to empirical findings only if they are statistically significant at the 5 percent level. We begin our analysis by exploring correlations among attribute ratings as well as correlations with the case outcome. In Table 3, we present a correlation table using the normalized ratings. As seen, the ratings are positively correlated across attributes, with *confident* and *aggressive* most correlated (*ρ* = 0.497) and *trustworthy* and *aggressive* least correlated (*ρ* = 0.102). Likewise, all attributes are positively correlated with the perceived likelihood of winning the case (e.g., advocates with voices perceived as more aggressive are also seen as more likely to win).

**Table 3:** Correlations in Case Outcome and Trait Judgements of Male Lawyers (N = 33,666). This table presents correlations in participant normalized ratings and case outcomes. Each observation is an argument by participant rating. *Case Outcome* is = 1 if advocate won the case, and = 0 if advocate lost. Bonferroni-adjusted *p*-values in parentheses.

Variable	Outcome	Aggressive	Attractive	Confident	Intelligent	Masculine	Trustworthy	Win
Case Outcome	1							
Aggressive	−0.00322	1						
	(1.000)							
Attractive	−0.00459	0.230**	1					
	(1.000)	(0.000)						
Confident	0.00243	0.497**	0.360**	1				
	(1.000)	(0.000)	(0.000)					
Intelligent	0.00814	0.235**	0.348**	0.401**	1			
	(1.000)	(0.000)	(0.000)	(0.000)				
Masculine	−0.0198**	0.345**	0.338**	0.442**	0.233**	1		
	(0.008)	(0.000)	(0.000)	(0.000)	(0.000)			
Trustworthy	−0.00541	0.102**	0.355**	0.266**	0.368**	0.200**	1	
	(1.000)	(0.000)	(0.000)	(0.000)	(0.000)	(0.000)		
Win	−0.00684	0.392**	0.439**	0.559**	0.477**	0.413**	0.397**	1
	(1.000)	(0.000)	(0.000)	(0.000)	(0.000)	(0.000)	(0.000)	

** indicates *p* < 0.01.

**Fig 2:**
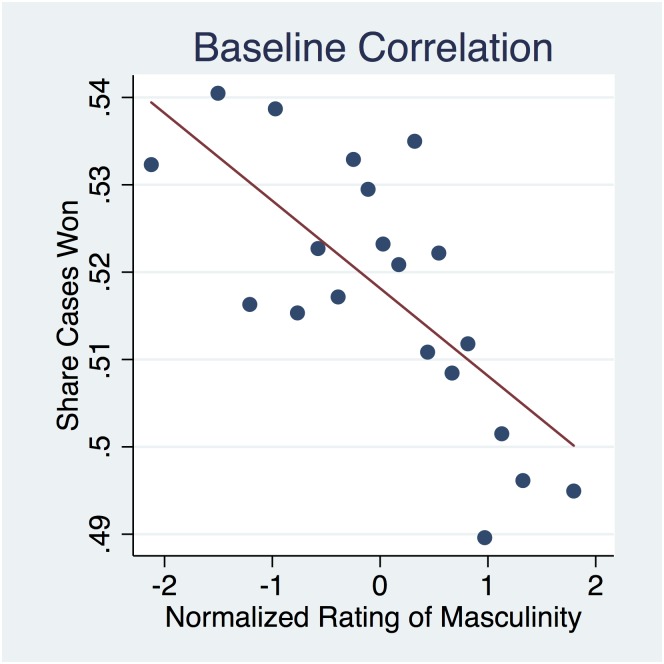
Advocate Masculinity and Court Outcomes. Binned scatterplots illustrating the association between voice-based masculinity ratings and court outcomes. Binned scatterplots are a non-parametric method of plotting the conditional expectation function (which describes the average y-value for each x-value). Ratings are sorted into twenty quantiles with each point in the figure indicating the share of oral arguments won for a given ratings bin. The figure reflects the correlation between normalized ratings of masculinity and case outcomes of male advocates.

In contrast, only masculinity is correlated with real outcomes (*ρ* = −0.02). To illustrate, we present a non-parametric plot of this correlation in Fig 2. In this figure, the normalized masculinity ratings are grouped into 20 equally sized bins with each point representing the share of cases won for observations in that bin. Notably, the slope between wins and masculinity is negative with a 5 percentage point difference in the likelihood of winning between advocates perceived as most and least masculine. We examine the robustness of this correlation in a regression framework that follows.

### Baseline Results

We begin by examining the relationship between voice-based perceptions of advocates and whether these perceptions can predict case outcomes. Focusing on our full sample of male advocates, the baseline results of estimating Eq (2) are presented in Table 4. As a starting point, we show OLS regression results using four different measures of attributes: normalized, untransformed, collapsed normalized and collapsed untransformed, where the collapsed measure is computed by collapsing the data to the mean attribute rating per audio clip. For each of these measures, we estimate two regression specifications, one excluding and one including lawyer fixed effects. The latter specification aims to approximate the relative correlation that stems from within-lawyer variation versus between-lawyer variation.

**Table 4:** OLS Baseline Results: Male Advocates. This table presents coefficient estimates from OLS regressions using data on Supreme Court oral arguments made by male advocates. The dependent variable is an indicator for whether the advocate won the case or not. Independent variables are voice-based ratings of advocate attributes made by survey participants, where untransformed ratings are integers ranging from 1 to 7 and normalized ratings are z-scored by participant. In columns 1-4, the unit of analysis is individual rating by oral argument, and in columns 5-8, the unit of analysis is oral argument average rating. Lawyer dummies are included where noted. Standard errors in parentheses are clustered by oral argument.

Dependent Variable: Case Outcome (= 1 if advocate won; = 0 if advocate lost)								
	Uncollapsed				Collapsed			
	(1)	(2)	(3)	(4)	(5)	(6)	(7)	(8)
	Normalized		Untransformed		Normalized		Untransformed	
Aggressive	−0.000718	−0.000359	0.00130	0.000292	−0.0324	−0.0397	0.00352	−0.0101
	(0.00423)	(0.00228)	(0.00270)	(0.00148)	(0.0550)	(0.127)	(0.0372)	(0.0831)
Attractive	−0.000228	0.00113	−0.00148	0.00187	−0.00894	−0.00646	−0.0248	0.0448
	(0.00488)	(0.00234)	(0.00342)	(0.00168)	(0.0465)	(0.126)	(0.0347)	(0.0881)
Confident	0.00738	0.00278	0.00426	0.00264	0.114^†^	0.0369	0.0547	0.0466
	(0.00466)	(0.00274)	(0.00306)	(0.00179)	(0.0671)	(0.152)	(0.0425)	(0.0969)
Intelligent	0.00747^†^	0.00333	0.00641*	0.000747	0.101^†^	0.0908	0.0846^†^	0.0118
	(0.00398)	(0.00214)	(0.00302)	(0.00162)	(0.0583)	(0.135)	(0.0452)	(0.106)
Masculine	−0.0122^†^	−0.00864**	−0.00978*	−0.00661**	−0.0315	−0.180^†^	−0.0316	−0.151*
	(0.00702)	(0.00305)	(0.00444)	(0.00198)	(0.0340)	(0.106)	(0.0237)	(0.0741)
Trustworthy	−0.00305	0.000746	−0.00105	0.00107	−0.0394	0.0245	−0.00585	0.0509
	(0.00351)	(0.00204)	(0.00276)	(0.00164)	(0.0576)	(0.125)	(0.0456)	(0.0950)
Win	−0.00451	0.00406	−0.00254	0.00274	−0.110	0.0920	−0.0752	0.0542
	(0.00425)	(0.00272)	(0.00325)	(0.00209)	(0.0718)	(0.143)	(0.0559)	(0.113)
Constant	0.518**	0.518**	0.529**	0.507**	0.518**	0.521**	0.453**	0.342
	(0.0124)	(0.00822)	(0.0285)	(0.0154)	(0.0124)	(0.0122)	(0.161)	(0.446)
Lawyer fixed effects	No	Yes	No	Yes	No	Yes	No	Yes
R squared	0.001	0.573	0.001	0.573	0.006	0.580	0.006	0.581
R squared Adj.	.0005315	.560479	.000677	.5605125	.0014215	.035004	.0020858	.0375119
Degrees of freedom	1633	1633	1633	1633	1633	1633	1633	1633
F statistic	1.384	1.858	1.375	2.138	1.325	0.789	1.469	0.907
Observations	33666	33666	33666	33666	1634	1634	1634	1634

^†^, *, and ** indicate significance at the 10 percent, 5 percent, and 1 percent levels, respectively.

Starting with columns (1)-(2) of Table 4, we show results using the normalized ratings. *Masculine* is significantly correlated with outcomes in the regression controlling for lawyer fixed effects. No other attributes are correlated with outcomes. The estimate from column (2) suggests that one standard deviation change in masculinity, for a given lawyer, is associated with a 0.9 percentage point change in case outcomes. Columns (3)-(4) repeat the same regressions using the untransformed scores, where each rating is an integer between 1 to 7. In the regression without lawyer fixed effects, both *intelligent* and *masculine* are correlated with case outcomes, but with the inclusion of lawyer fixed effects only *masculine* remains significant. Since the standard deviation of *masculine* using the untransformed scores is approximately 1.5, the correlation magnitudes are comparable to those obtained using the normalized scores. Running this set of regressions with the collapsed data yields little further insight. The only significant coefficient in columns (5)-(8) is the one on *masculine* in column (8), the specification using the collapsed untransformed measures with lawyer fixed effects.

Taken together, in half the regression specifications there is evidence for a correlation between *masculine* and outcomes. This partial pattern motivates further inquiry. As for the other attributes, we find no correlations except for intelligence in 1 of the 8 regressions. Likewise, participants are poor at predicting court outcomes based on the voice stimuli alone.

### Petitioners versus Respondents

Under a hypothesis of the primacy of first impressions on court decisions, the first person to argue in front of the Justices should exhibit a stronger vocal first impression effect. That is, the first speaker may have a longer lasting impact on the court and subsequent outcomes, a hypothesis we derive from the anchoring effect [46], where individuals rely on an initial piece of information to make subsequent judgments. As the advocates for the petitioner always argue before the advocates for the respondent at the Supreme Court, we examine the robustness of the association between perceived masculinity and court outcomes separately for the petitioners and respondents. We report the results of our analysis in Table 5.

**Table 5:** OLS Results: Male Petitioners versus Respondents. This table presents coefficient estimates from OLS regressions using data on Supreme Court oral arguments made by male advocates. Columns 1-5 (6-10) use data on oral arguments made by advocates for the petitioner (respondent). The dependent variable is an indicator for whether the advocate won the case or not. Independent variables are voice-based ratings of advocate attributes normalized by survey particiapnt. Lawyer and participant dummies are included where noted. Participant controls are age and dummies for each category given in the biographical questionnaire. Standard errors in parentheses are clustered by oral argument.

Dependent Variable: Case Outcome (= 1 if advocate won; = 0 if advocate lost)										
	Petitioners					Respondents				
	(1)	(2)	(3)	(4)	(5)	(6)	(7)	(8)	(9)	(10)
Aggressive	−0.00496	−0.00552	0.00282	0.00280	0.00278	0.00116	0.00112	−0.00430	−0.00429	−0.00374
	(0.00538)	(0.00533)	(0.00263)	(0.00263)	(0.00272)	(0.00590)	(0.00591)	(0.00304)	(0.00303)	(0.00304)
Attractive	−0.00103	−0.000832	−0.0000731	−0.000188	−0.000559	−0.000221	−0.00156	0.00328	0.00300	0.00248
	(0.00638)	(0.00624)	(0.00269)	(0.00270)	(0.00275)	(0.00676)	(0.00675)	(0.00319)	(0.00321)	(0.00323)
Confident	0.00912	0.00947	0.00393	0.00396	0.00417	−0.00985	−0.00949	−0.00642^†^	−0.00642^†^	−0.00658^†^
	(0.00584)	(0.00580)	(0.00299)	(0.00299)	(0.00302)	(0.00664)	(0.00658)	(0.00341)	(0.00344)	(0.00347)
Intelligent	0.00736	0.00601	0.00284	0.00291	0.00247	0.00399	0.00521	0.000153	−0.000169	0.000370
	(0.00538)	(0.00527)	(0.00239)	(0.00239)	(0.00243)	(0.00561)	(0.00555)	(0.00274)	(0.00275)	(0.00274)
Masculine	−0.0197*	−0.0211*	−0.00727*	−0.00766*	−0.00765*	0.00731	0.00778	0.00141	0.00136	0.00182
	(0.00904)	(0.00885)	(0.00364)	(0.00363)	(0.00359)	(0.00962)	(0.00944)	(0.00365)	(0.00366)	(0.00361)
Trustworthy	0.00166	0.00157	0.00176	0.00166	0.00169	−0.00965^†^	−0.00943^†^	−0.00277	−0.00270	−0.00252
	(0.00447)	(0.00438)	(0.00234)	(0.00233)	(0.00232)	(0.00508)	(0.00505)	(0.00272)	(0.00271)	(0.00270)
Win	−0.00270	−0.00153	0.00270	0.00294	0.00290	−0.0145*	−0.0144*	−0.000903	−0.000927	−0.00110
	(0.00536)	(0.00520)	(0.00292)	(0.00289)	(0.00295)	(0.00602)	(0.00591)	(0.00343)	(0.00344)	(0.00340)
Constant	0.669**	0.669**	0.669**	0.745^†^	0.681**	0.350**	0.350**	0.351**	0.206	0.316**
	(0.0162)	(0.0157)	(0.00988)	(0.447)	(0.0479)	(0.0172)	(0.0167)	(0.0105)	(0.470)	(0.0524)
Fixed effects	No	Participant	Lawyer	Lawyer	Lawyer & participant	No	Participant	Lawyer	Lawyer	Lawyer & participant
Participant controls	No	No	No	Yes	No	No	No	No	Yes	No
R squared	0.002	0.047	0.634	0.636	0.649	0.002	0.047	0.639	0.641	0.654
R squared Adj.	.0013846	.0130435	.6232579	.6237974	.6252639	.0017658	.0092158	.6270483	.6270729	.6285589
Degrees of freedom	855	855	855	855	855	777	777	777	777	777
F statistic	1.256	1.407	1.374	1.387	1.187	2.082	2.116	1.132	1.372	1.443
Observations	17665	17665	17665	17665	7665	16001	16001	16001	16001	16001

^†^, *, and ** indicate significance at the 10 percent, 5 percent, and 1 percent levels, respectively.

**Fig 3:**
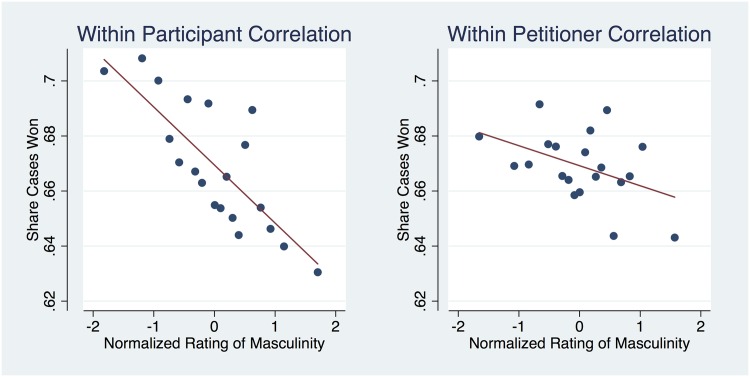
Petitioner Masculinity and Court Outcomes. Binned scatterplots illustrating the association between voice-based masculinity rating and court outcomes. Binned scatterplots are a non-parametric method of plotting the conditional expectation function (which describes the average y-value for each x-value). The figures are residual plots of the regressions presented in columns 2 (left) and 3 (right) of Table 5, excluding the *masculine* independent variable. The lefthand (righthand) side figure plots residuals net of survey participant (lawyer) dummies. Ratings are sorted into twenty quantiles with each point in the figure indicating the mean residual for a given ratings bin.

Indeed, the key observation is that the correlation between perceived masculinity and outcomes persists for petitioners but not for respondents. The correlation is robust across multiple specifications using a combination of participant controls and lawyer and participant fixed effects. Focusing on the subsample of arguments made by advocates for petitioners, in column (1) of Table 5, the baseline regression, the coefficient estimate suggests a 2 percentage points increase in case wins associated with one standard deviation decrease in *masculine.* In column (2), we show that this estimate is robust to the inclusion of participant fixed effects. This means that the correlation between perceived masculinity and real outcomes is not driven by any subset of survey participants. Put differently, this specification excludes cross-participant variation in ratings, such that the results are driven solely by variation in participant ratings of the random set of 66 audio clips. In column (3), we examine the correlation within-lawyer by including lawyer fixed effects. The estimate on *masculine* is 0.007 suggesting that about 1/3 of the correlation between masculinity and court outcomes is driven by variation in oral arguments made by the same advocate versus 2/3 that is driven by variation in arguments made by different advocates. To illustrate these last results, we provide a nonparametric plot of the residuals obtained from regressing case outcomes on the set of fixed effects and attributes excluding *masculine* against the masculine ratings. In Fig 3, we provide the residual plots reflecting columns (2) and (3). For example, the lefthand side plot, which parallels the within-participant regression in column (2), shows a difference of approximately 8 percentage points in winning between oral arguments made by advocates perceived as least and most masculine.

To control for the possibility that participants with certain characteristics are driving the results, we further expand our analysis by including participant characteristics. Specifically, we include controls for participant age, and dummies for each racial group, gender, income cohort, education level and state of residence (see Table 1). Column (4) in Table 5 presents regression results that includes this set of participant controls in addition to lawyer fixed effects, column (5) substitutes these participant controls with participant fixed effects. Point estimates on *masculine* remain similar and significant in these specifications. No other coefficient estimates of attributes are significant in this set of petitioner regressions.

Turning to the respondent regressions, we do not find any of the attributes to be correlated with case outcomes. To the extent that we do find significant results, these are limited to the two regression specifications that leverage between-advocate variation (columns (1)-(2)), where perceptions of winning are negatively correlated with actually winning. We do not focus on these results, given that this correlation (a) is not specific to an attribute, (b) does not persist across regression specifications and (c) does not have support in the baseline regressions presented in Table 4. We also find no further support in these regressions for intelligence as a possible correlate of outcomes.

To sum, we find robust evidence for a correlation between case outcomes and voice-based perceptions of advocate masculinity for petitioners. No association between perceived masculinity and court outcomes is found among lawyers of the respondents. This finding supports the hypothesis that first impression, in this case, of the first lawyer to argue before the Justices, exhibits a disproportionate association with judicial decisions.

## Robustness and Extensions

In this section, we expand our analysis in a number of directions, including robustness to sample, ratings, and model variations.

Given our findings that, even once removing cross-advocate variation, the negative correlation between perceptions of masculinity and court outcomes persists, we examine more closely whether our results are driven by cases argued in a certain year or by advocates with a certain degree of experience in arguing cases at the Supreme Court. To do this, we compare our baseline regression results for petitioners (column (1) in Table 5) to the regression results in Table 6. By including year fixed effects, column (1) in Table 6 addresses whether our findings are driven by a certain set of cases in our sample of oral arguments. Similarly, column (2) includes fixed effects for the number of oral arguments in our sample made by the same lawyer, which we take as a proxy for experience. In both specifications, the estimate on *masculine* remains significant but is slightly smaller in magnitude (1.7 versus 2 percentage points in the baseline regression). Given this, we can rule out that cohort, or time effects are significantly influencing our findings.

**Table 6:** Robustness Checks: Male Petitioners. This table presents coefficient estimates from regressions using data on Supreme Court oral arguments made by male advocates for the petitioner. The dependent variable is an indicator for whether the advocate won the case or not. Independent variables are voice-based ratings of advocate attributes normalized by survey particiapnt. Columns 1-2 report coefficient estimates using OLS with dummies for year of argument and number of cases argued by the lawyer where noted. Columns 3-4 report coefficient estimates using OLS where ratings that exceed the Mahalanobis distance of $\sqrt{\chi_{6,0.975}^{2}}=3.801$ are omitted in column 3, and ratings by survey participants with scores in the top quintile on a measure of rating inconsistency are omitted in column 4 (see S1 Table). Columns 5-6 report baseline probit (logistic) regression results with marginal effects calculated at the means of the independent variables. Standard errors in parentheses are clustered by oral argument.

Dependent Variable: Case Outcome (= 1 if advocate won; = 0 if advocate lost)								
Advocate Appearance			Participant Ratings			Estimation Method		
	(1)	(2)		(3)	(4)		(5)	(6)
Aggressive	−0.00667	−0.00404	Aggressive	−0.00385	−0.00392	Aggressive	−0.00495	−0.00497
	(0.00519)	(0.00519)		(0.00599)	(0.00572)		(0.00540)	(0.00538)
Attractive	−0.000999	0.000381	Attractive	−0.00284	0.000871	Attractive	−0.00101	−0.00105
	(0.00614)	(0.00591)		(0.00687)	(0.00675)		(0.00640)	(0.00639)
Confident	0.00822	0.00602	Confident	0.0117^†^	0.00636	Confident	0.00919	0.00916
	(0.00583)	(0.00571)		(0.00699)	(0.00659)		(0.00587)	(0.00585)
Intelligent	0.00635	0.00722	Intelligent	0.0101^†^	0.00480	Intelligent	0.00728	0.00737
	(0.00535)	(0.00513)		(0.00603)	(0.00597)		(0.00537)	(0.00537)
Masculine	−0.0177*	−0.0166*	Masculine	−0.0221*	−0.0204*	Masculine	−0.0197*	−0.0198*
	(0.00874)	(0.00834)		(0.00964)	(0.00964)		(0.00911)	(0.00914)
Trustworthy	0.00185	0.00172	Trustworthy	0.00464	0.00202	Trustworthy	0.00169	0.00166
	(0.00438)	(0.00433)		(0.00510)	(0.00479)		(0.00448)	(0.00448)
Win	−0.00266	−0.00282	Win	−0.00730	−0.00213	Win	−0.00272	−0.00271
	(0.00525)	(0.00514)		(0.00633)	(0.00588)		(0.00539)	(0.00538)
Fixed effects	Year of Case	Number of Cases	Excluded ratings	MD Outliers	Inconsistent Raters	Regression model	Probit	Logistic
R squared	0.032	0.053	R squared	0.002	0.002	Pseudo R squared	0.001	0.001
F statistic	1.223	1.003	F statistic	1.644	0.890	Chi squared	8.717	8.708
Observations	17665	17665	Observations	14913	14290	Observations	17665	17665

^†^ and * indicate significance at the 10 percent and 5 percent levels respectively.

We next examine how our results change if we remove ratings that can be deemed as outliers. The first method to identify such outliers is by computing the Mahalanobis distance (MD) for ratings given by each participant for each audio clip. We then run the baseline regression excluding ratings that exceed the critical value associated with a 2.5 percent significance level, about 15 percent of our ratings. Column (3) in Table 6 shows the regression results excluding these ratings. The estimate on *masculine* is significant and slightly larger: one standard deviation increase in masculinity is associated with 2.2 percentage points decrease in winning. A second method we use to identify outliers is based on examining ratings on the set of 6 repeat audio clips. For each participant we computed a consistency score defined as the average absolute difference in attribute ratings on the set of identical audio clips. The mean (and median) consistency score across participants and attributes is approximately one (further details are available in S1 Table). In column (4), we present regression results excluding ratings by a 1/5 of participants with the worst consistency scores. As seen, the association between perceived masculinity and outcomes remains similar to the one in the baseline regression. We take these results to indicate that our findings are likely to be stronger if we were to carefully screen out ratings by participants who may have misunderstood or exerted insufficient effort on the task.

In the final set of regressions, we show that our baseline estimates are robust to estimation method. In columns (5) and (6) of Table 6, we report estimates of marginal effects derived from applying a probit and logistic regression, respectively. In both cases, the estimate on *masculine* is nearly identical to the one we obtained using OLS.

To examine whether the ratings we gathered are specific to our procedure, we varied the survey design on a subsample of 60 voice clips. Instead of the basic design where the listener is presented with one voice sample and rates the sample on all attributes, the participants were randomly assigned to rate only one attribute for each recording, thus obviating the potential of cross-attribute influence on each other for a given voice clip and also to control for the possibility of within-voice modeling by participants. The key difference between this survey and our main survey depicted in Fig 1 is that only one attribute, selected at random for each voice recording, appeared in question 1. While there are slight differences in ratings across surveys, the results are very similar suggesting further robustness of our key findings on the connection between voice-based trait judgments of advocates and Supreme Court outcomes. We illustrate the high degree of correlation in perceptions across surveys (S1 Fig) in the Supplemental Information (SI).

Likewise, for this same subsample of 60 voice clips we were able to collect detailed information regarding the biographical characteristics of the advocates. Specifically, these include age, law school, whether the advocate was a member of the law review, had an additional graduate degree, was a Supreme Court clerk, and the total number of clerkships the advocate had. We found that including these covariates in a regression increased the precision of the estimate on *masculine* (see S2 Table). Overall, we acknowledge that we are unable to make far reaching conclusions from these regressions given the small sample size; however, if perceptions of masculinity were simply reflecting other important advocate covariates, then the coefficient estimates on *masculine* should be driven to zero. That this is not the case suggests that the channel of how trait judgments stemming from an extremely brief voice clip predict outcomes may not be as simple as one might expect. Likewise, our results are unlikely to be driven by any specific choice of number of ratings or survey framing. In sum, these findings are unlikely to be driven by spurious correlations or measurement error and provide further credence to the notion that snap judgments that stem from even 3-second voice samples can influence listeners beliefs about those they face and subsequent actions.

Finally, it is worth noting that only about 15 percent of the advocates who argued in the Supreme Court during the time period of our study were female. The gender-specificity of our findings is a question that warrants further investigation, especially since studies on voice-based social biases observed significant differences in how listeners react to voices of different perceived gender [47]. However, due to the lack of statistical power, we leave this question for future studies with an expanded female advocate dataset. Relatedly, we explored whether perceptions differ by gender of survey participant and whether such differences could affect how the perceived attributes of male advocates predict case outcomes. While we found some differences in ratings (most notably, female participants, more than male, perceive masculine advocates as more intelligent), we did not find these to play a role in our key finding on the relationship between voice-based perceptions of masculinity and outcomes in the Supreme Court.

## Discussion

To the best of our knowledge, this is the first study documenting an association between voice-based impressionistic judgments and judicial decisions. To benchmark our findings, the 2 percentage point difference in court outcomes attributed to one standard deviation change in perceived masculinity is equivalent to more than 1/2 of the gender gap (i.e., in our sample, male lawyers are 3.7 percentage points more likely to win a court case than female lawyers). These associations are comparable to effects of other external factors that have been shown to influence judicial behavior. For example, asylum judges are 2 percentage points more likely to deny asylum to refugees if their previous decision granted asylum [48]. Likewise, asylum judges are roughly 2 percentage points more likely to grant asylum on the day after a home-city Sunday football game win instead of a loss [49]. In a similar vein, U.S. District judges are a 0.3 percentage point less likely to assign any prison length in criminal sentencing cases after a home-city football game win instead of a loss [49]. More generally, judges’ demographic background characteristics, such as gender, race, and in particular, party of appointing president [50, 51], especially before elections [52], have all been shown to correlate with their decision-making over a range of legal issues.

Our findings echo earlier research documenting associations between voice-based personality judgments and human behavior. For instance, previous studies have found vocal attractiveness to be an important social evaluation linked to mate selection and sexual behavior [35] and masculine voices to be linked to dominance [31] and men’s threat potential in forager and industrial societies [53]. This type of association extends beyond evolutionary implications and may affect immediate real world consequences. Perceived intelligence, for example, has been found to affect an individual’s employability [28]. Landlords are found to discriminate against prospective tenants on the basis of the sound of their voice during telephone conversations [11]. Perceived task-ability, dominance, and sociability are found to show the strongest correlation with perceived influence in simulated juries [12]. Thus, the association between voice-based personality and court outcomes observed in this study further strengthens the importance of understanding how (and why) voice-based judgments influence human behavior.

To be sure, what is still in need of further exploration is the specific nature of the association between voice judgments and court outcomes. That is, why are court outcomes correlated with perceived masculinity but not other attributes? It is worth noting that the focus on language and gender in the court room is not new. However, previous studies have focused primarily on the gendered language performance of witnesses [54] or the discursive practices in the courtroom [55]. To the best of our knowledge, no studies have focused on vocal characteristics of the lawyers *per se*. More specifically, given that the attributes are positively correlated with each other, the fact that only perceived masculinity is found to correlate with court outcomes suggests that masculinity captures particular variance that is not captured by the other ratings. In a similar study where subjects were presented with faces of electoral candidates and were asked to rate the candidates’ perceived attributes, such as competence, intelligence, leadership, honesty, trustworthiness, charisma, and likability of candidates [33], only perceptions of competence predicted election outcomes. Our findings are similar in that, while perceived masculinity correlated with judgments of other voice attributes, perceived masculinity is the only one that predicts court outcomes in a consistent and robust manner.

Concerning the nature of the perceived attribute itself, masculinity is a quality or set of practices that is stereotypically, though not exclusively, connected with men. Women may engage in masculine practices equally as much, although such practices are either not noticed or censured [56]. The performative nature of “masculinity” made possible the existence of non-masculine men and masculine women [56–59]. Different cultures may also construct different notions of masculinity. These differences are reflected in the stereotypical ways of talking and thinking about men and masculinities. In the US, there are four main cultural discourses of masculinity [56]: gender difference, which pertains to categorical difference in biology and behavior between men and women; heterosexism, which sees being masculine as to sexually desire women and not men; dominance, which links masculinity with notions of authority or power; and male solidarity, which assumes as given a bond among men.

In the present context, the fact that court outcomes are negatively associated with masculinity points to a possible connection with the discourse of dominance. That is, lawyers who are perceived to be more masculine might be construed as being more dominant and authoritative. To what extent these constructs, as distinct from perceived confidence and perceived aggressiveness, play a role in the decision process as judges deliberate court decisions will have to be explored further in future work. This work only establishes an association and does not attempt to advocate a particular causal relationship between these variables. To be sure, gendered differentiation of masculine and feminine language has been argued to have different evolutionary basis [60]. Males are seen as being selected to be aggressive and dominant, but this selective pressure might be a double-edged sword since aggressive and dominant behaviors would lead to lethal confrontation. In the present context, the dominant and aggressive stands of masculine-sounding lawyers might have invited an adverse response from the Court.

Given our research design, our findings do not allow us to conclude if the Justices were engaging in some form of linguistic profiling in making their judicial decision *per se*. Do lawyers change their voices across oral arguments in a manner predicted by case characteristics? Do law firms engage in some form of linguistic profiling in choosing their oral advocates? Further investigation should yield fruitful insights into the mechanisms underlying the associations between voice-based masculinity and court outcomes.

In sum, our results contribute to a growing literature on the relevance of extraneous factors in courtrooms. That is, although judicial behavior is widely assumed to be governed by legal doctrine [61], where judges are strictly hewing to legal doctrine and court precedent in making their decisions, the judge’s decision can be affected by the judge’s policy preferences [62], self-interest [63], and in the present case, potential voice-based snap judgments regarding lawyer personality. Future studies will hopefully elucidate the mechanisms behind these extraneous factors in the courtroom.

## Supporting Information

S1 Fig Correlation in Ratings across Survey Designs (collapsed). This figure plots the mean untransformed rating for each of the 60 audio clips selected from our sample for further robustness checks. The x-axis reflects mean ratings obtained from participants in our main survey who were asked to rate each advocate on the full set of attributes, whereas the y-axis reflects the mean ratings obtained from participants in an alternative survey who were randomly assigned to rate each advocate on only one attribute at a time.
(TIFF)

S2 Fig First Screenshot of Survey. (TIF)

S3 Fig Second Screenshot of Survey. (TIF)

S4 Fig Third Screenshot of Survey. (TIF)

S1 TableParticipant Ratings Consistency (N = 748).This table presents descriptive statistics of a measure of consistency in participant ratings using data on the random set of 6 audio clips that were duplicated for each participant. For each participant, the consistency measure is defined as the averge absolute difference in ratings of a given attribute between the duplicate clips: $abs(rating{}_{itw}-rating_{itw}^{\prime })/2$.(PDF)

S2 Table Robustness Checks on Sample of 60 Clips. This table presents coefficient estimates from OLS regressions using data on a select sample of Supreme Court oral arguments made by male advocates. The dependent variable is an indicator for whether the advocate won the case or not. Independent variables are voice-based ratings of advocate attributes normalized by survey participant. Column 1 reports basline regression results, column 2 reports results from a specification that includes lawyer biographical controls: age, number of clerkships, and dummies for whether the advocate attended an elite law school, has a second graduate degree, served on law review or as a Supreme Court clerk. Columns 3-4 compare regression results using alternative survey designs to the baseline results presented in column 1. Column 3 presents results from a survey of approximately 200 participants rating the set of 60 audio clips, and column 4 presents results using ratings obtained from a survey that randomly assigned only one attribute to each audio clip. ^*a*^ ratings of educatedness were included instead of aggressiveness in columns 3-4; ^*b*^ ratings of age were included instead of intelligence in column 4; †, *, and ** indicate significance at the 10 percent, 5 percent, and 1 percent levels, respectively.
(PDF)
