# Supplementary figures and images for: Perceived Masculinity Predicts U.S. Supreme Court Outcomes
Source: PLoS One. 2016 Oct 13;11(10):e0164324. doi: 10.1371/journal.pone.0164324 (PMC5063312; doi:10.1371/journal.pone.0164324)

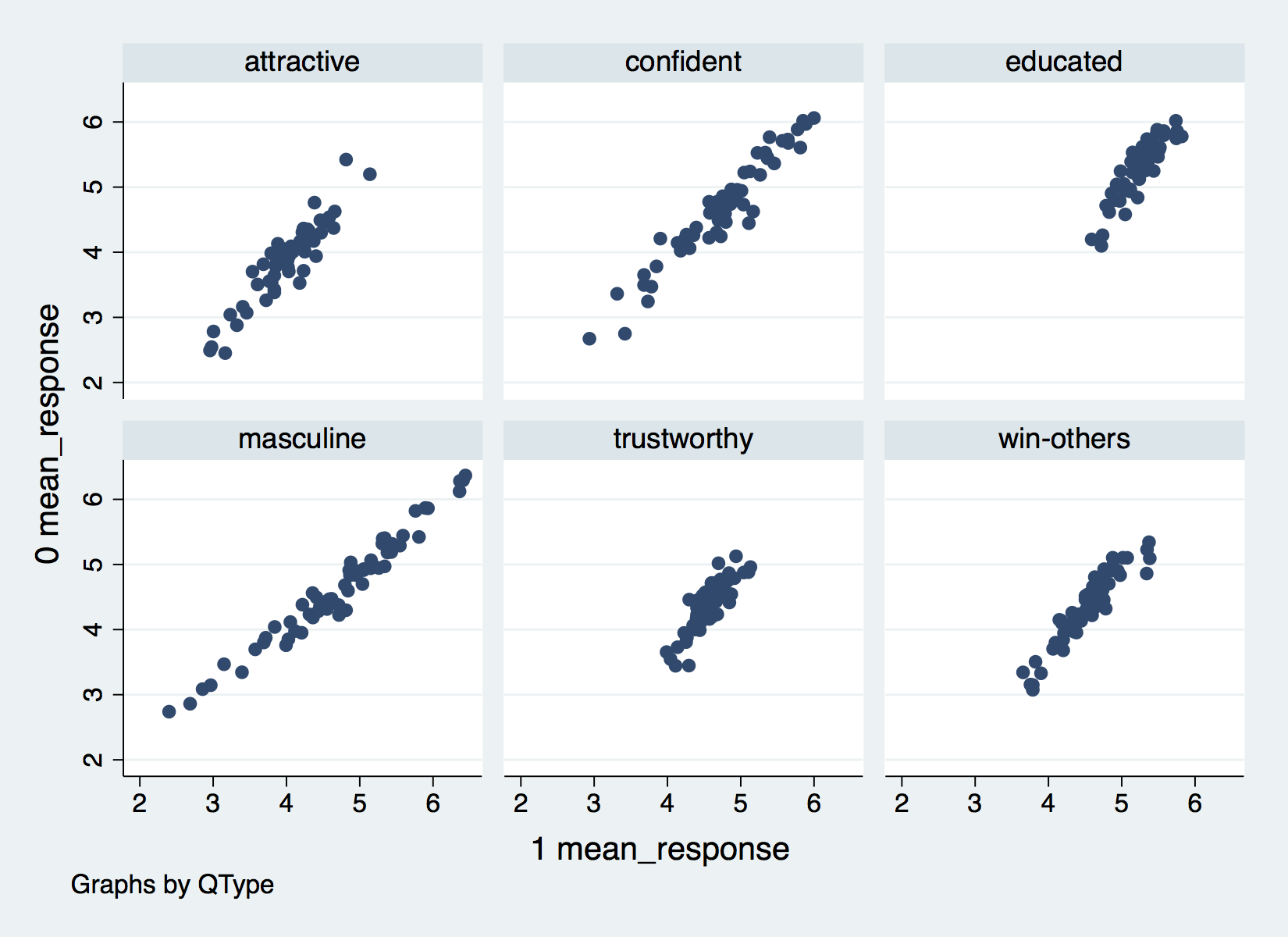

Supplement: S1 Fig — This figure plots the mean untransformed rating for each of the 60 audio clips selected from our sample for further robustness checks. The x-axis reflects mean ratings obtained from participants in our main survey who were asked to rate each advocate on the full set of attributes, whereas the y-axis reflects the mean ratings obtained from participants in an alternative survey who were randomly assigned to rate each advocate on only one attribute at a time. (TIFF) [file pone.0164324.s001.tiff]

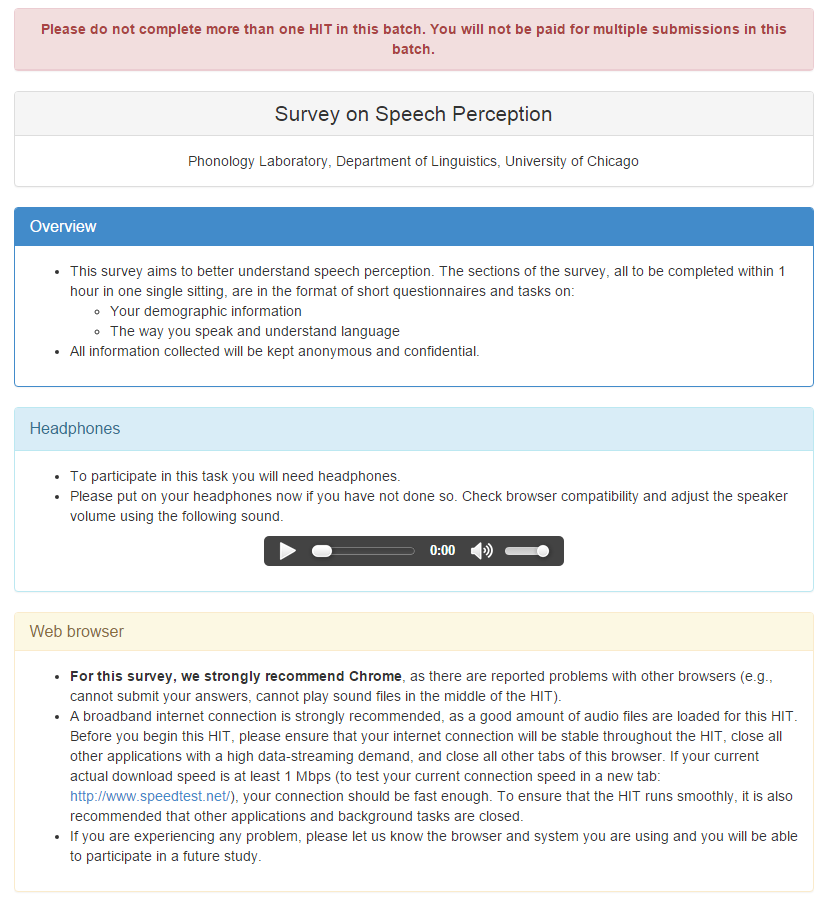

Supplement: S2 Fig — (TIF) [file pone.0164324.s002.tif]

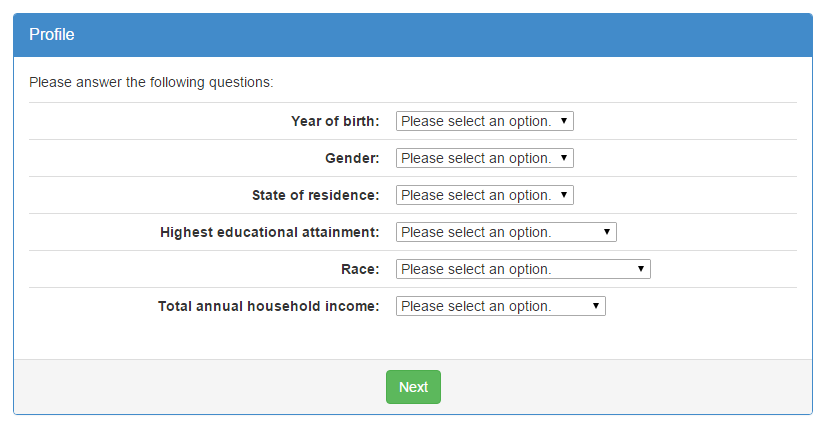

Supplement: S3 Fig — (TIF) [file pone.0164324.s003.tif]

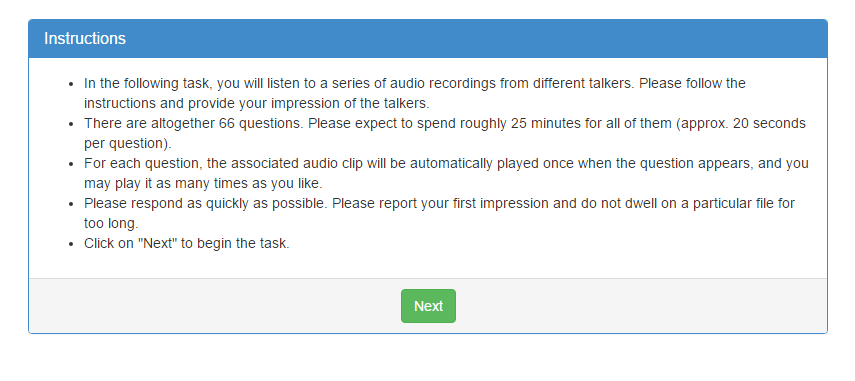

Supplement: S4 Fig — (TIF) [file pone.0164324.s004.tif]
