# Supplementary material for: Perceived Masculinity Predicts U.S. Supreme Court Outcomes
Source: PLoS One. 2016 Oct 13;11(10):e0164324. doi: 10.1371/journal.pone.0164324 (PMC5063312; doi:10.1371/journal.pone.0164324)
Supplement: S1 Table — This table presents descriptive statistics of a measure of consistency in participant ratings using data on the random set of 6 audio clips that were duplicated for each participant. For each participant, the consistency measure is defined as the averge absolute difference in ratings of a given attribute between the duplicate clips: abs(ratingitw-ratingitw′)/2. (PDF) [file pone.0164324.s005.pdf]

S1 Table. Participant Ratings Consistency (N = 748). This table presents descriptive statistics of a measure of consistency in participant ratings using data on the random set of 6 audio clips that were duplicated for each participant. For each participant, the consistency measure is defined as the averge absolute difference in ratings of a given attribute between the duplicate clips : $abs\left(rating_{itw} - rating'_{itw}\right)/2$.

	Mean	10th Percentile	25th Percentile	Median	75th Percentile	90th Percentile
Aggressive	1.053922	.3333333	.6666667	1	1.333333	1.833333
Attractive	.8805704	.1666667	.5	.8333333	1.166667	1.5
Confident	1.096702	.5	.6666667	1	1.333333	1.833333
Intelligent	.8573975	.1666667	.5	.8333333	1.166667	1.5
Masculine	.9193405	.3333333	.5	.8333333	1.166667	1.666667
Trust	.9021836	.1666667	.5	.8333333	1.166667	1.666667
Win	.9146613	.3333333	.5	.8333333	1.166667	1.666667
