# Supplementary material for: Perceived Masculinity Predicts U.S. Supreme Court Outcomes
Source: PLoS One. 2016 Oct 13;11(10):e0164324. doi: 10.1371/journal.pone.0164324 (PMC5063312; doi:10.1371/journal.pone.0164324)
Supplement: S2 Table — This table presents coefficient estimates from OLS regressions using data on a select sample of Supreme Court oral arguments made by male advocates. The dependent variable is an indicator for whether the advocate won the case or not. Independent variables are voice-based ratings of advocate attributes normalized by survey participant. Column 1 reports basline regression results, column 2 reports results from a specification that includes lawyer biographical controls: age, number of clerkships, and dummies for whether the advocate attended an elite law school, has a second graduate degree, served on law review or as a Supreme Court clerk. Columns 3-4 compare regression results using alternative survey designs to the baseline results presented in column 1. Column 3 presents results from a survey of approximately 200 participants rating the set of 60 audio clips, and column 4 presents results using ratings obtained from a survey that randomly assigned only one attribute to each audio clip. a ratings of educatedness were included instead of aggressiveness in columns 3-4; b ratings of age were included instead of intelligence in column 4; †, *, and ** indicate significance at the 10 percent, 5 percent, and 1 percent levels, respectively. (PDF) [file pone.0164324.s006.pdf]

S2 Table. Robustness Checks on Sample of 60 Clips. This table presents coefficient estimates from OLS regressions using data on a select sample of Supreme Court oral arguments made by male advocates. The dependent variable is an indicator for whether the advocate won the case or not. Independent variables are voice-based ratings of advocate attributes normalized by survey participant. Column 1 reports basline regression results, column 2 reports results from a specification that includes lawyer biographical controls: age, number of clerkships, and dummies for whether the advocate attended an elite law school, has a second graduate degree, served on law review or as a Supreme Court clerk. Columns 3-4 compare regression results using alternative survey designs to the baseline results presented in column 1. Column 3 presents results from a survey of approximately 200 participants rating the set of 60 audio clips, and column 4 presents results using ratings obtained from a survey that randomly assigned only one attribute to each audio clip. [a] ratings of educatedness were included instead of aggressiveness in columns 3-4; [b] ratings of age were included instead of intelligence in column 4; †, *, and ** indicate significance at the 10 percent, 5 percent, and 1 percent levels, respectively.

Dependent Variable: Case Outcome (= 1 if advocate won; = 0 if advocate lost)

	Baseline<br>(1)	Advocate Biography<br>(2)	Tenfold Ratings<br>(3)	Single Attribute<br>(4)
Aggressive[a]	0.0171	0.0254		
	(0.0224)	(0.0200)		
Attractive	0.00950	0.00544	-0.0142	-0.0208
	(0.0230)	(0.0193)	(0.0216)	(0.0219)
Confident	0.00376	0.00575	0.0254	-0.0132
	(0.0239)	(0.0190)	(0.0263)	(0.0238)
Intelligent[b]	0.0274	0.0242	0.00463	
	(0.0194)	(0.0154)	(0.00896)	
Masculine	-0.0567†	-0.0644*	-0.0982*	-0.0541†
	(0.0310)	(0.0263)	(0.0381)	(0.0297)
Trustworthy	0.0205	0.0205	-0.00485	-0.0135
	(0.0170)	(0.0145)	(0.00874)	(0.0140)
Win	-0.0181	-0.0225	-0.00192	-0.00391
	(0.0197)	(0.0168)	(0.00855)	(0.0198)
Constant	0.505**	0.388	0.500**	0.502**
	(0.0664)	(0.354)	(0.0633)	(0.0643)
R squared	0.013	0.134	0.033	0.018
R squared Adj.	.0076092	.1239723	.0319564	.0168711
Degrees of freedom	57	57	59	59
F statistic	1.160	3.379	1.655	0.859
Observations	1184	1184	11040	4631
